# In-season assessment of agronomic nitrogen use efficiency and its components in winter wheat using critical nitrogen dilution curve

**DOI:** 10.3389/fpls.2023.1128799

**Published:** 2023-03-15

**Authors:** Bing Li, Xiaodong Ma, Ben Zhao, Laigang Wang, Syed Tahir Ata-Ul-Karim

**Affiliations:** ^1^ International School, Huanghe Science & Technology College, Zhengzhou, Henan, China; ^2^ Farmland Irrigation Research Institute, Chinese Academy of Agricultural Sciences, Xinxiang, Henan, China; ^3^ Graduate School of Agricultural and Life Sciences, The University of Tokyo, Tokyo, Japan

**Keywords:** accumulated nitrogen deficit, nitrogen diagnosis, nitrogen fertilizer scheduling, plant nitrogen concentration, winter wheat

## Abstract

Accurate and timely nitrogen (N) scheduling requires knowledge of in-season crop N deficit. Therefore, understanding the association between crop growth and crop N demand during its growth period is imperative for fine-tuning N scheduling decisions to actual crop N demand and to enhance N use efficiency. The concept of the critical N dilution curve has been employed to assess and quantify the intensity and time of crop N deficit. However, research regarding the association between crop N deficit and N use efficiency in wheat is limited. The present study was carried out to determine whether there are relationships between the accumulated nitrogen deficit (N_and_) and agronomic N use efficiency (AE_N_) as well as with its components (N fertilizer recovery efficiency (RE_N_) and N fertilizer physiological efficiency (PE_N_)) of winter wheat and to explore the potential capacity of N_and_ for predicting AE_N_ and its components. Data acquired from five variable N rates (0, 75, 150, 225, and 300 kg ha^−1^) field experiments using six winter wheat cultivars were used to establish and validate the relationships between N_and_ and AE_N_, RE_N_, and PE_N_. The results indicated that plant N concentration in winter wheat was significantly affected by N application rates. N_and_ varied from −65.73 to 104.37 kg ha^−1^ after Feekes stage 6 under different N application rates. The AE_N_ and its components were also affected by cultivars, N levels, seasons, and growth stages. A positive correlation was observed between N_and_, AE_N_, and its components. Validation using an independent data set showed the robustness of the newly developed empirical models to accurately predict AE_N_, RE_N,_ and PE_N_ with an RMSE of 3.43 kg kg^−1^, 4.22%, and 3.67 kg kg^−1^ and RRMSE of 17.53%, 12.46%, and 13.17%, respectively. This indicates that N_and_ has the potential to predict AE_N_ and its components during the growth period of winter wheat. The findings will assist in improving in-season N use efficiency by fine-tuning N scheduling decisions in winter wheat cultivation.

## 1 Introduction

Wheat is grown in nearly every region of the world. The economic and dietary significance of wheat for million of smallholder farmers,poorest, and undernourished people cannot be disputed. Therefore, wheat is imperative for ensuring global food and nutritional security (Zheng et al., 2021). Wheat production has kept pace with the gigantic increase in human population, mainly due to high-yield cultivars and N fertilizer application. N fertilizer is the most important plant nutrient for enhancing the grain yield and quality of major cereals (Ata-Ul-Karim et al., 2016; Zhao et al., 2021). However, inappropriate N fertilizer application not only decreases the agronomic N use efficiency (AE_N_) but also poses negative side effects, disturbing aquatic and terrestrial ecosystems as well as seriously afflicting the atmosphere (Rutting et al., 2018). Therefore, it is critical to optimize the current N use in crop production to improve the AE_N_ and reduce the N requirement.

The critical N dilution curve is a plant-based diagnostic approach that has been widely used for over three decades for diagnosing crop N status. Additionally, it has also been integrated as a reference index for calibrating other methods of crop N management such as chlorophyll meters, remote sensing, and soil nitrate (Devienne-Barret et al., 2000; Zhao et al., 2016; Zha et al., 2020). A critical N dilution curve-based N nutrition index has been widely used to assess crop N status. Accumulated N deficit (N_and_) is also a critical N dilution curve-based index, which also has the potential to quantify crop N status (Ata-Ul-Karim et al., 2013, Zhao et al., 2022). The critical N dilution curve for winter wheat has been established in different countries around the world, including China (Yue et al., 2012). N_and_, being a crop-specific, precise, and theoretically sound index in relation to actual crop growth, is agronomically relevant to serve as an N diagnostic tool (Ata-Ul-Karim et al., 2013).

Nitrogen is required by plants for generating a photosynthetically active canopy to ensure optimal grain yield and storing enough grain protein. Crop growth, grain yield, and grain quality are highly dependent on substantial N inputs. N application in agricultural production has markedly increased over the past half-century worldwide, including in China (Wang and Lu, 2020). China is currently the largest consumer of N fertilizer worldwide. Excessive N application is a common practice in the intensive cropping systems of China. The average N application rates in wheat-producing regions of China reach up to 550 kg ha^−1^ year^−1^ (Zhang et al., 2015). Excessive N use has led to low AE_N_ (27.5%) in wheat-growing regions (Ding et al., 2018). Agronomists and crop breeders are trying to seek crop cultivars with better AE_N_ to ensure higher grain yields with low N requirements. Previous studies showed that AE_N_ could be affected by N fertilizer application (Halvorson et al., 2005; Zhai et al., 2019). Therefore, it is important to understand the changes in AE_N_ due to N fertilizer application to develop cost-effective and eco-friendly N management strategies. The AE_N_ is crucial to interpreting and understanding the trade-offs between optimal agriculture production and the potential loss of N fertilizer. Additionally, the variability in AE_N_ can also assist in the adoption of suitable crop management practices by growers as well as the selection of wheat cultivars with higher N use efficiencies (Cantarella et al., 2018). It is challenging to compare the difference in N fertilizer use under different N management conditions in agricultural production without prior knowledge of AE_N_ (Sadras and Denison, 2016). Therefore, understanding the limited factors related to AE_N_ is essential to improving crop production and reducing N loss. This study hypothesizes that the crop N deficit has a significant effect on the values of AEN and its components across different N treatments, there is a strong linear relationship between N_and_ and AE_N_ and that this relationship can be employed for in-season assessment of crop N status. However, little has been done to investigate the relationship of N_and_ with AE_N_ and its components during the growth period of winter wheat.

Therefore, this study aims to develop the relationship of N_and_ with AE_N_ and its components (physiological N fertilizer efficiency (PE_N_) and N fertilizer recovery efficiency (RE_N_)) for winter wheat. The findings will assist in improving in-season N use efficiency by fine-tuning N scheduling decisions in winter wheat cultivation.

## 2 Materials and methods

### 2.1 Experimental site and design

**Figure 1:**
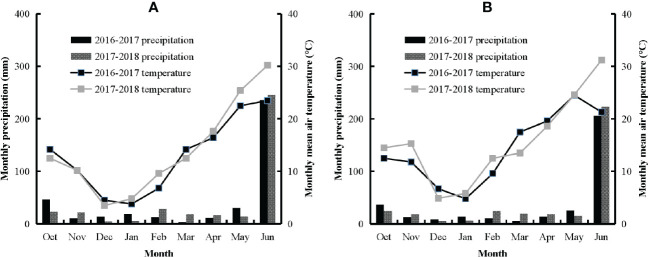
Monthly mean air temperature (°C) and precipitation (mm) from 2016 to 2018 seasons at different experimental sites **(A)** Xinxiang (XX); **(B)** Jiaozuo (JZ).

Four multi-locational experiments with varied N application rates were conducted at Xinxiang and Jiaozuo from 2016 to 2018 using the six most widely grown winter wheat cultivars in the region. The detailed description of soil characteristics of experimental sites, cultivars, N rates, planting, sampling, and harvesting timing is shown in **Table 1**. The weather conditions during the experimental period are shown in **Figure 1**. Treatments were replicated thrice using a randomized complete block design. The size of each plot was 24 m^2^ (6 m × 4 m). The seeding rate was 240 kg ha^−1^. N fertilizer (urea, 46% N) was applied (50:50) as basal and top dressing before sowing and at Feekes stage 4. P (as P_2_O_5_) and K (as K_2_O) were applied at the rates of 120 kg ha^−1^ and 105 kg ha^−1^, respectively. The broadcasting method of fertilizer application was used to apply fertilizer to each plot. Weeds, pests, and diseases were managed using chemical methods, with chemicals applied at recommended rates. Each plot was irrigated evenly using surface irrigation with a 4-inch plastic-coated hose. N fertilizer application was the only factor limiting crop growth and productivity.

**Table 1:** Characteristics of the four field experiments conducted in this study during 2016–2018.

Experiment/Location	Season	Soil characteristics (20cm)	Cultivar	Sowing date	Harvest date	N rate (kg ha^−1^)	Sampling stage
Experiment 1	2016/2017	Type: sandy soil	Zhoumai22 (ZM22)	14 October	3 June	0 (N0)	Feekes 6 (Stem elongation)
Xinxiang		Organic matter 13.4 g kg^−1^	Zhoumai27 (ZM27)			75 (N75)	Feekes10 (Booting)
		Total N 1.1 g kg^−1^				150 (N150)	Feekes 10.51 (Anthesis)
		Available P 62 mg g^−1^				225 (N225)	Feekes 11.4 (Filling)
		Available K 75.5 mg g^−1^				300 (N300)	
Experiment 2	2016/2017	Type: sandy soil	ZM22	12 October	6 June	0 (N0)	Feekes 6
Jiaozuo		Organic matter 11.23 g kg^−1^	ZM27			75 (N75)	Feekes 10
		Total N 1.4 g kg^−1^				150 (N150)	Feekes 10.51
		Available P 12.14 mg g^−1^				225 (N225)	Feekes 11.4
		Available K 131 mg g^−1^				300 (N300)	
Experiment 3	2017/2018	Type: sandy soil	Bainong207 (BN207)	16 October	5 June	0 (N0)	Feekes 6
Xinxiang		Organic matter 16.26 g kg^−1^				90 (N90)	Feekes 10
		Total N 1.1 g kg^−1^				180 (N180)	Feekes 10.51
		Available P 44 mg kg^−1^				270 (N270)	Feekes 11.4
		Available K 80 mg kg^−1^					
Experiment 4	2017/2018	Type: sandy soil	Aikang58 (AK58)	12 October	2 June	0 (N0)	Feekes 6
Jiaozuo		Organic matter 10.14 g kg^−1^	Yumai58 (YM58)			75 (N75)	Feekes 10
		Total N 0.8 g kg^−1^	Xinmai26 (XM26)			150 (N150)	Feekes 10.51
		Available P 13.44 mg kg^−1^				225 (N225)	Feekes 11.4
		Available K 141 mg kg^−1^					

### 2.2 Crop sampling and measurements

Twenty plants were sampled from each experimental plot at different Feekes stages for the determination of plant dry matter (DM) and plant N concentration (PNC). Plant samples were oven-dried for 48 h at 80°C to get a constant weight and then weighed on an analytical balance, followed by grinding and sieving through a sieve for lab analysis. Samples of 0.15 g of ground plant biomass were digested using a mixture of H_2_O_2_ and H_2_SO_4_, and the PNC was determined using the Kjeldahl method (Bremner and Mulvancy, 1982).

Grain yield at maturity was determined by harvesting a 1 m^2^ area from each plot and adjusting the moisture content to 14%. A thousand grain weight (TGW) was measured from 1,000 grain samples. Grain protein content was measured by multiplying the grain N concentration by a factor of 6.25, while protein yield was measured by multiplying crop yield by grain protein content. The descriptions, abbreviations, and units of all indices are described in **Supplementary Table S1**.

### 2.3 Accumulated N deficit

Plant N_and_ was estimated by subtracting the critical plant N accumulation (PNA) from the actual PNA across different N rates at each sampling stage (Ata-Ul-Karim et al., 2013). The dilution curve of N_c_ concentration (N_c_ = 4.15DM^−0.38^) of winter wheat was used to calculate the value of N_and_ (Yue et al., 2012).


(1) $$N_{cna}=\;10N_{c}\times DM$$

(2) $$N_{and}=\;N_{cna}-\;N_{na}$$

Where N_cna_ is the PNA under the N_c_ condition (kg ha^−1^), N_na_ is the actual PNA across varied N (kg ha^−1^), and 10 is the conversion factor. If N_and_ is equal to 0, optimal N nutrition is observed. If N_and_ = 0, N nutrition was considered optimum, while N_and_ >0 indicated N deficiency and v<0 pointed out luxury consumption (Ata-Ul-Karim et al., 2013).

### 2.4 Agronomic nitrogen use efficiency and its components

Agronomic N use efficiency is the amount of additional grain harvested per kilogram of N fertilizer applied. AE_N_ could be represented as G_w_/N_f_, where G_w_ is grain yield and N_f_ is N fertilizer application (kg ha^−1^) (Halvorson et al., 2005). This is defined as (G_wi_ − G_w0_)/N_f_ where i is the level of N fertilizer under various treatments and 0 is the N fertilizer under control treatment.

The two primary components of AE_N_ are: (1) N fertilizer recovery efficiency (RE_N_), which describes the recovery efficiency of fertilizer N from soil (N_ti_ − N_t0_)/N_f_ (Zhang et al., 2008), and (2) physiological N use efficiency (PE_N_) is the measure of the ability of a plant to produce grain/biomass with N acquired at the whole plant level (G_wi_ − G_w0_)/(N_ti_ − N_t0_) (Zhang et al., 2008), where N_t_ represents plant N accumulation at harvest. Therefore, AE_N_ was expressed by the following equation:


(3) $$AE_{N}=\frac{G_{wi}-G_{w0}}{N_{f}}=\frac{N_{t0}-N_{t0}}{N_{f}}\times \frac{G_{wi}-G_{w0}}{N_{ti}-N_{t0}}$$

### 2.5 Data analysis

The data of PNC, grain yield, grain protein content, grain protein yield, TGW, AE_N_, RE_N_, and PE_N_ were subjected to one-way analysis of variance analysis for comparing the statistical difference using the SPSS software package version 22 (SPSS Inc., Chicago, IL, USA). The difference between the data means was assessed using the least significant difference (LSD) test at the 5% level. The fixed effects included cultivar, N treatments, and site, while the random effects included block. The fitted linear model was estimated based on the least square method.

### 2.6 Development and validation of models

Data acquired from field experiments in 2016–2017 were used to develop the relationship between N_and_ and AE_N_ and its components, while the data acquired in 2017–2018 were used to validate the newly developed relationships. Root mean square error (RMSE), relative root mean square error (RRMSE), and a 1:1 plot were used to determine the robustness of newly developed models between the estimated and observed values of AE_N_, RE_N_, and PE_N_. RMSE and RRMSE were calculated as follows:


(4) $$\text{RMSE=}\sqrt{\frac{\sum_{i=1}^{\text{n}}{{\text{(P}}_{\text{i}}{\text{-O}}_{\text{i}})}^{2}}{\text{n}}}$$

(5) $$\text{RMSE=}\frac{RMSE}{\tilde{O}}\times 100\%$$

where n represents the sample number, and P_i_ and O_i_ represent estimated and observed values, respectively. Õ is the average value of the observed values.

## 3 Results

### 3.1 Plant nitrogen concentration under different nitrogen rates

**Figure 2:**
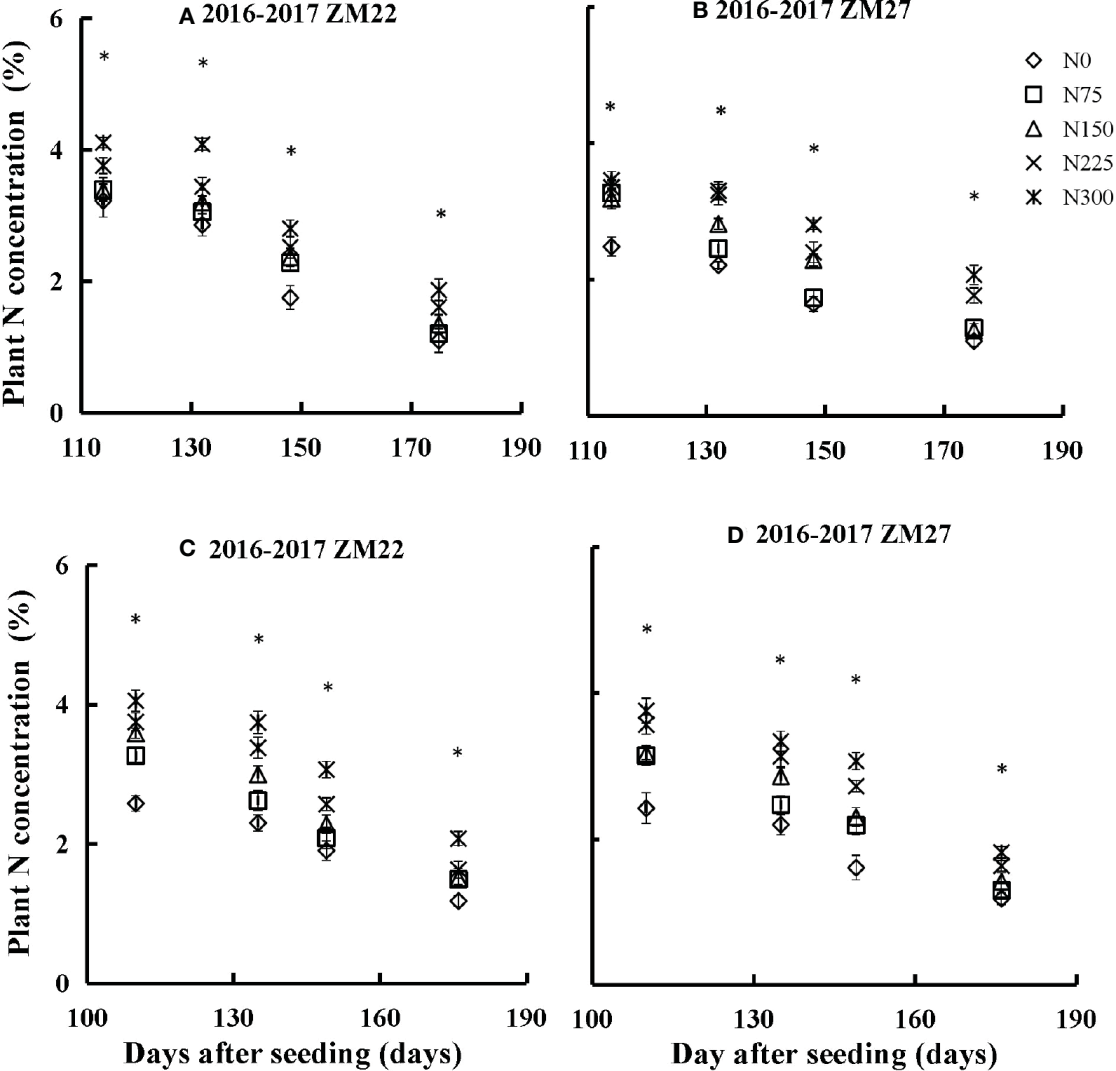
Changes in PNC as a function of time (days after sowing) at various N rates in the 2016–2017 seasons and for different cultivars **(A, B)** Xinxiang; **(C, D)** Jiaozuo. The vertical bar represents the standard deviation of plant N concentration, and the symbol *indicates that the *p*-value is<0.05.

Plant N concentration increased with the increase in N input rates, and a significant difference was observed for PNC under different N treatments and growth stages (**Figure 2**). PNC significantly declined from Feekes stage 6 to Feekes stage 11.4 with the growth of winter wheat during the 2016–2017 season at Xinxiang and Jiaozuo (**Supplementary Tables S2, S3**). The maximum value of PNC was observed at Feekes stage 6 for both cultivars, while the minimum value of PNC was observed at Feekes stage 11.4. PNC ranged from 1.19% to 4.18% and 1.09% to 3.46% for ZM22 and ZM27, respectively, during the 2016–2017 season of Xinxiang (**Figures 2A, B**), while PNC during the 2016–2017 season of Jiaozuo ranged from 1.18% to 4.05% and 1.18% to 3.76% for ZM22 and ZM27, respectively (**Figures 2C, D**). Overall, ZM22 showed higher PNC than ZM27 across different N treatments, growth stages, seasons, and experimental sites.

### 3.2 Grain yield and quality-related indices under different nitrogen rates

Grain yield, grain protein content, grain protein yield, and TGW were not affected (*p<*0.05) by the interactive effect of cultivar × N rate, cultivar × site, N rate × site, and cultivar × N rate × site. The grain yield of both cultivars was affected by the application of N fertilizer (**Table 2**), and maximum grain yield was observed with ZM27. ZM27 showed 10% higher grain yield than ZM22. The grain yield of both cultivars declined under maximum N application (300 kg ha^−1^) during the 2016–2017 wheat growing season. Higher grain protein yield and grain protein content were observed for ZM22 as compared to ZM27. Grain protein yield and protein content of ZM22 were 20% and 8% higher than those of ZM27, respectively. Besides, grain protein yield and grain protein content were also affected by N application. Grain protein yield and grain protein content of both cultivars showed an average increase of 19% and 137%, respectively, as compared with the N0 treatment. However, little variation was observed in grain protein yield and grain protein content across the sites. Grain protein content was slightly higher at Xinxiang than at Jiaozuo, while grain protein yield was higher at Jiaozuo. Additionally, the TGW of both cultivars was not affected by fertilizer application rates.

**Table 2:** Mean values of yield, protein content, protein yield, and a thousand grain weight (TGW) of two cultivars under five N levels during the 2016–2017 growing seasons.

Treatments	Yield (kg ha^−1^)	Protein content (%)	Protein yield (kg ha^−1^)	TGW (g)
Cultivar (C)				
ZM22	5,033.88 ± 123.56b	14.56 ± 0.56a	738.85 ± 11.5a	35.8 ± 2.6a
ZM27	5,522.34 ± 131.56a	12.13 ± 0.34b	681.28 ± 20.56b	36.7 ± 1.85a
Nitrogen (N) rate				
N0	3,217.88 ± 85.63d	12.18 ± 0.54c	391.94 ± 18.43d	35.6 ± 2.58a
N75	4,835.47 ± 102.56c	13.45 ± 0.48b	650.37 ± 22.54c	36.7 ± 1.57a
N150	5,809.54 ± 75.42b	14.18 ± 0.68ab	823.79 ± 20.23b	37.9 ± 3.56a
N225	6,300.79 ± 83.48a	14.62 ± 0.42a	921.18 ± 17.85a	38.2 ± 3.21a
N300	6,226.88 ± 120.35a	14.89 ± 0.35a	927.18 ± 14.12a	37.8 ± 2.85a
Site (S)				
Xinxiang	5,411.11 ± 123.5a	13.56 ± 0.72a	717.81 ± 14.25a	36.1 ± 2.47a
Jiaozuo	5,145.11 ± 147.56b	13.48 ± 0.53a	729.41 ± 12.34a	35.4 ± 3.05a
Interaction				
C × N	ns	ns	ns	ns
C × S	ns	ns	ns	ns
N × S	ns	ns	ns	ns
C × N × S	ns	ns	ns	ns

The different letters between columns in the same row are significantly different (p<0.05), ns refers to no significant difference at 0.05 level.

### 3.3 Accumulated nitrogen deficit under different nitrogen rates

**Figure 3:**
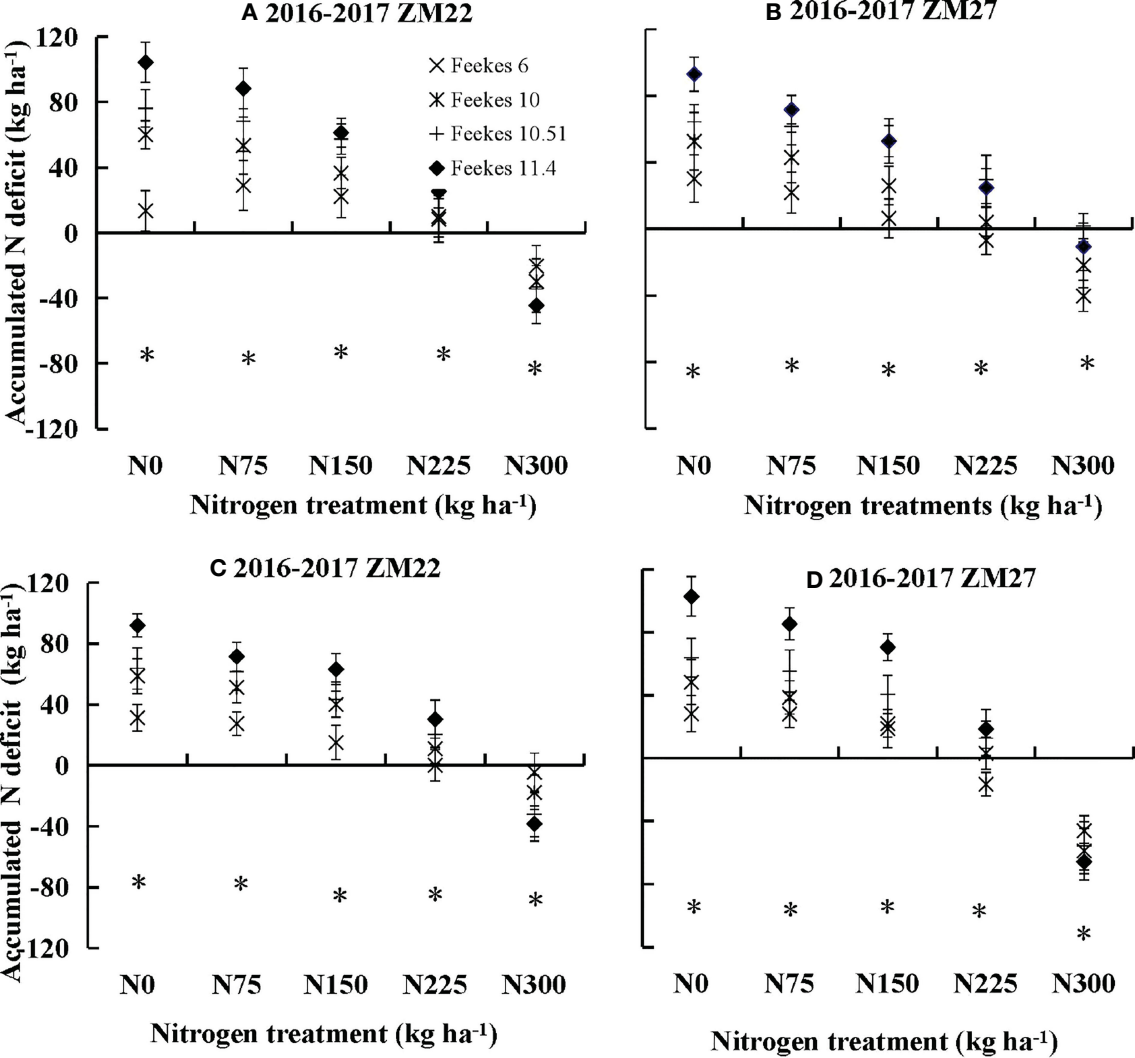
Changes in accumulated N deficit for winter wheat under different N application rates in experiments conducted from 2016 to 2017 **(A, B)** Xinxiang; **(C, D)** Jiaozuo. The vertical bar represents the standard deviation of accumulation N deficit, and the symbol *indicates that the *p*-value is<0.05.

The accumulated nitrogen deficit varied from −65.73 to 104.37 kg ha^−1^ under different N rates across seasons, cultivars, sites, and growth stages of winter wheat. There were substantial differences in N_and_ across different N treatments and cultivars (**Figure 3**). The N_and_ showed a declining trend with increasing N application rates, while it increased gradually towards advancing maturity. This increase towards advancing maturity was obvious under N-limiting treatments (N0, N75, and N150), and it reached its maximum value at Feekes stage 11.4. Conversely, this increase was minor under optimal N conditions (N225), while under non-N-limiting treatment (N300), excess N accumulation was observed until Feekes stage 11.4. The N_and_ ranged from 104.37 to −44.42 kg ha^−1^ and 93.07 to −40.64 kg ha^−1^ during the 2016 to 2017 season at Xinxiang for ZM22 and ZM27, respectively, while it ranged from 91.97 to −38.27 kg ha^−1^ and 102.08 to −65.73 kg ha^−1^ during the 2016 to 2017 season at Jiaozuo for ZM22 and ZM27, respectively. Non-significant differences were observed for N_and_ across different cultivars and sites (*p*-value = 0.817). The N_and_ values were higher than 0 for N0, N75, and N150 treatments, were almost equal to 0 for the N225 treatment, and were lower than 0 for the N300 treatment across sites and seasons.

### 3.4 Agronomic nitrogen fertilizer use efficiency and its components under different nitrogen rates

Agronomic N fertilizer use efficiency and its components (RE_N_ and PE_N_) were not affected (*p<*0.05) by the interactive effect of cultivar × N rate, cultivar × site, N rate × site, and cultivar × N rate × site. AE_N,_ RE_N,_ and PE_N_ were affected by N fertilizer application (**Table 3**). The values of AE_N_, PE_N,_ and RE_N_ ranged from 12.5 to 23.56 kg kg^−1^, 23.51 to 37.39 kg kg^−1^, and 31.6% to 55.7%, respectively, across different N rates during the 2016 to 2017 season (**Table 3**). AE_N_, RE_N,_ and PE_N_ were higher at 75 kg N ha^−1^ than those at 300 kg N ha^−1^, which indicates that AE_N_, RE_N,_ and PE_N_ declined with increasing N supply. ZM27 showed higher values of AE_N_ and PE_N_ than ZM22. The ZM27 of AE_N_ and PE_N_ were 42% and 37.6% higher than those of ZM22, while the RE_N_ of ZM22 was 5% lower than that of ZM27. However, non-significant differences were observed for AE_N_, RE_N,_ and PE_N_ of wheat cultivars at both sites; the minor differences might be attributed to experimental error (**Table 3**).

**Table 3:** **N** fertilizer agronomic efficiency and its components for two cultivars under different N levels during the 2016 to 2017 growing seasons and the mean values of the two cultivars under the different N levels.

Treatments	AE_N_ (kg kg^−1^)	RE_N_ (%)	PE_N_ (kg kg^−1^)
Cultivar (C)			
ZM22	13.95 ± 3.39b	43.6 ± 3.5a	30.12 ± 2.91b
ZM27	19.82 ± 2.56a	41.4 ± 4.2a	41.45 ± 6.06a
Nitrogen (N) rate			
N (75-0)	23.56 ± 7.09a	55.7 ± 6a	37.39 ± 2.83a
N (150-0)	18.97 ± 3.57ab	49.5 ± 6ab	32.94 ± 3.59b
N (225-0)	15.95 ± 2.14bc	41.8 ± 10bc	28.81 ± 2.08bc
N (300-0)	12.5 ± 1.87c	31.6 ± 6c	23.51 ± 4.5c
Site (S)			
Xinxiang	17.85 ± 6.09a	43.7 ± 14a	28.73 ± 8.43a
Jiaozuo	16.11 ± 5.95a	41.8 ± 11a	23.44 ± 5.94a
Interaction			
C × N	ns	ns	ns
C × S	ns	ns	ns
N × S	ns	ns	ns
C × N × S	ns	ns	ns

The different letters between columns in the same row are significantly different (p<0.05), ns refers to no significant difference at 0.05 level.

### 3.5 Relationships between accumulated nitrogen deficit and agronomic nitrogen use efficiency and its components

**Figure 4:**
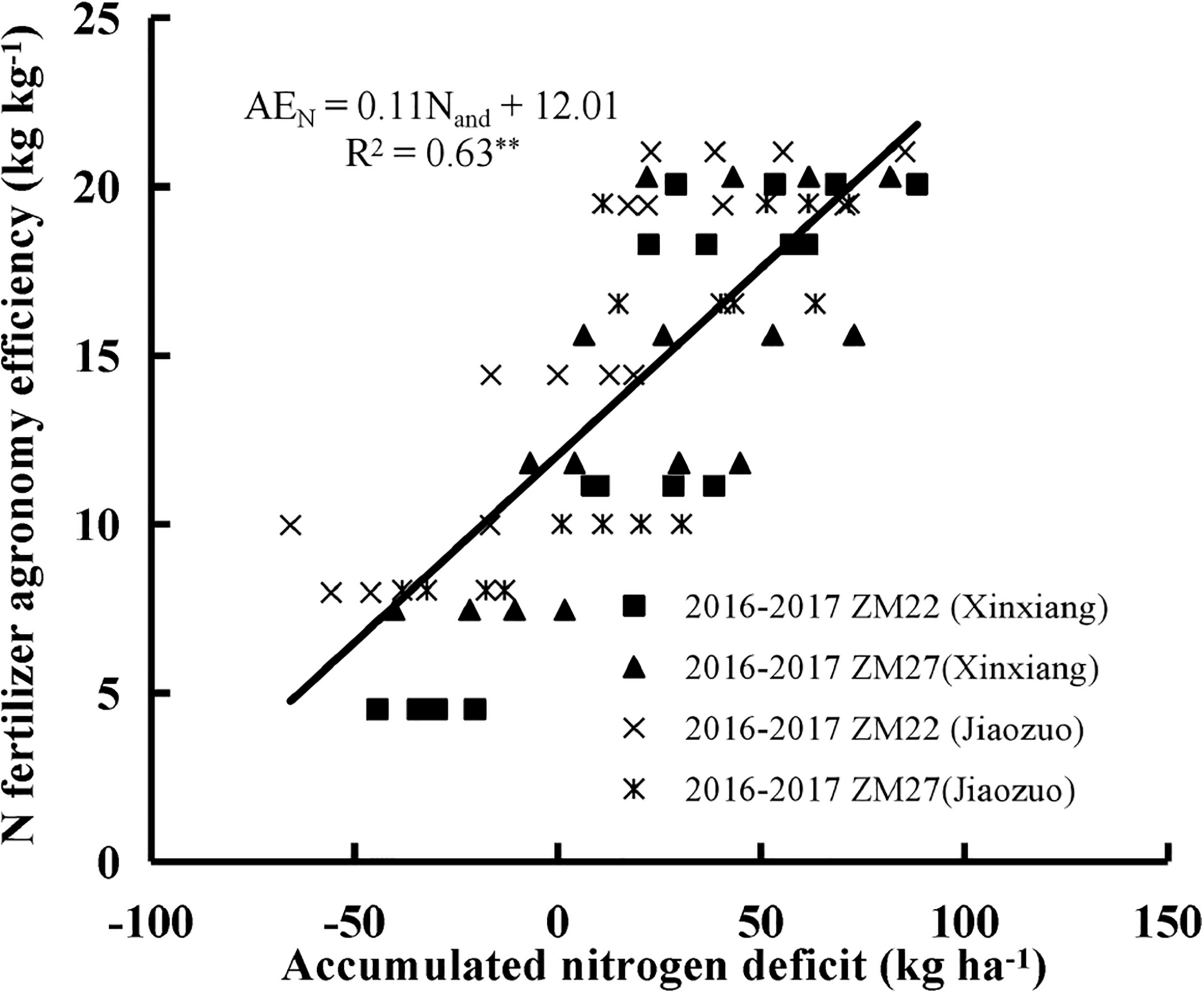
The relationship between accumulated N deficit and N fertilizer agronomic efficiency of winter wheat from 2016 to 2017 at Xinxiang and Jiaozuo. The symbol **indicates that the *p*-value is<0.01.

**Figure 5:**
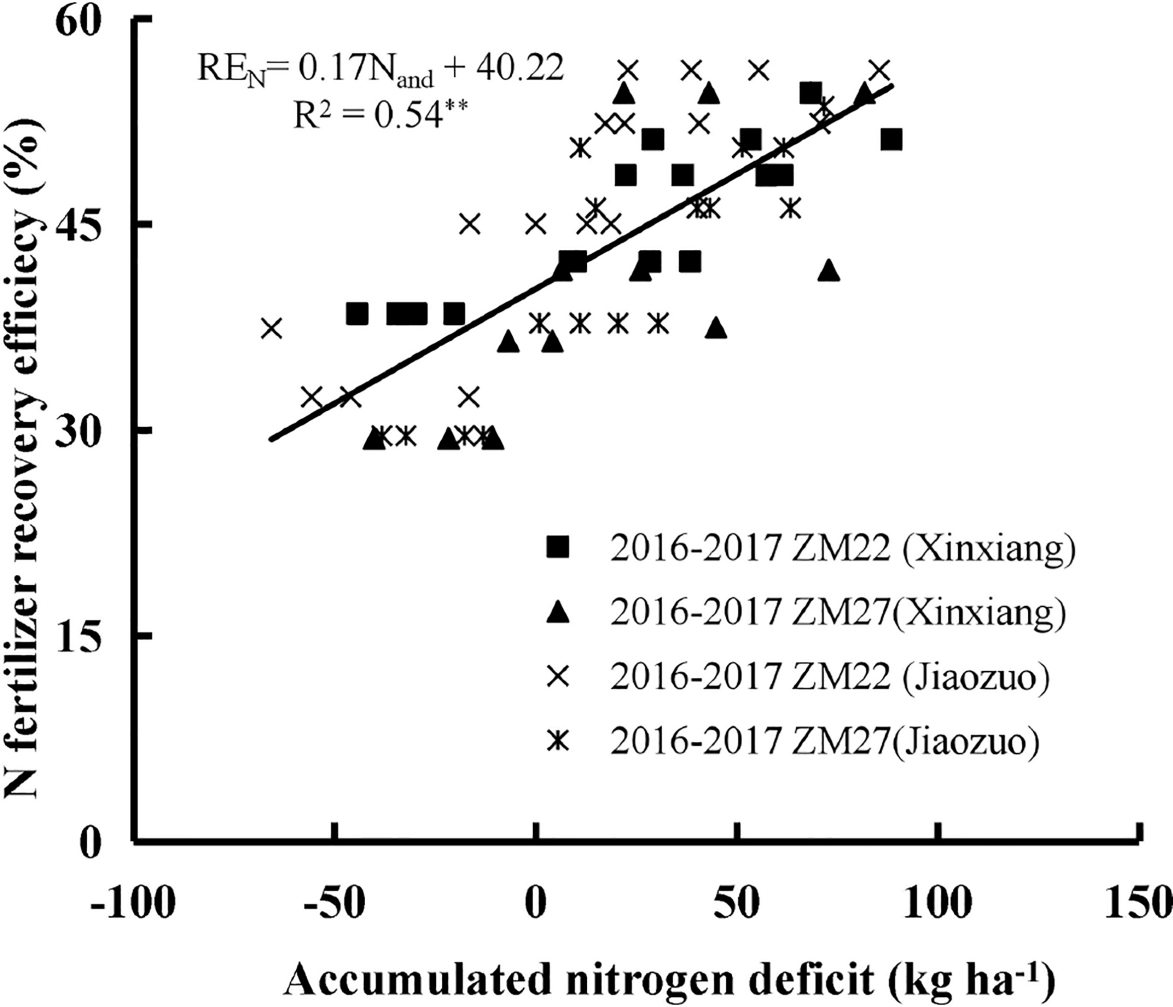
The relationship between accumulated N deficit and N fertilizer recovery efficiency of winter wheat from 2016 to 2017 at Xinxiang and Jiaozuo. The symbol **indicates that the *p*-value is<0.01.

**Figure 6:**
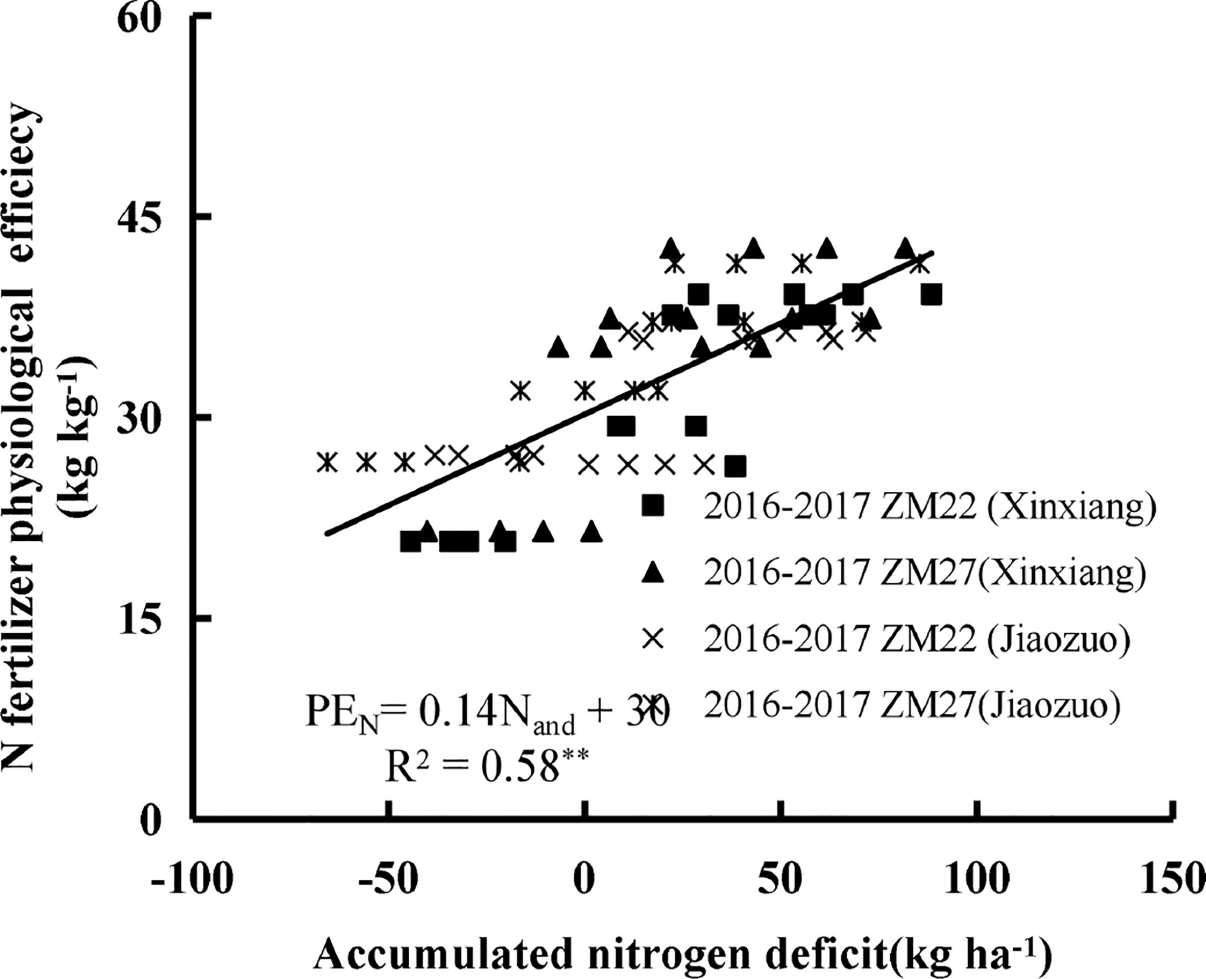
The relationship between accumulated N deficit and N fertilizer physiological efficiency of winter wheat from 2016 to 2017 at Xinxiang and Jiaozuo. The symbol **indicates that the *p*-value is<0.01.

**Figure 7:**
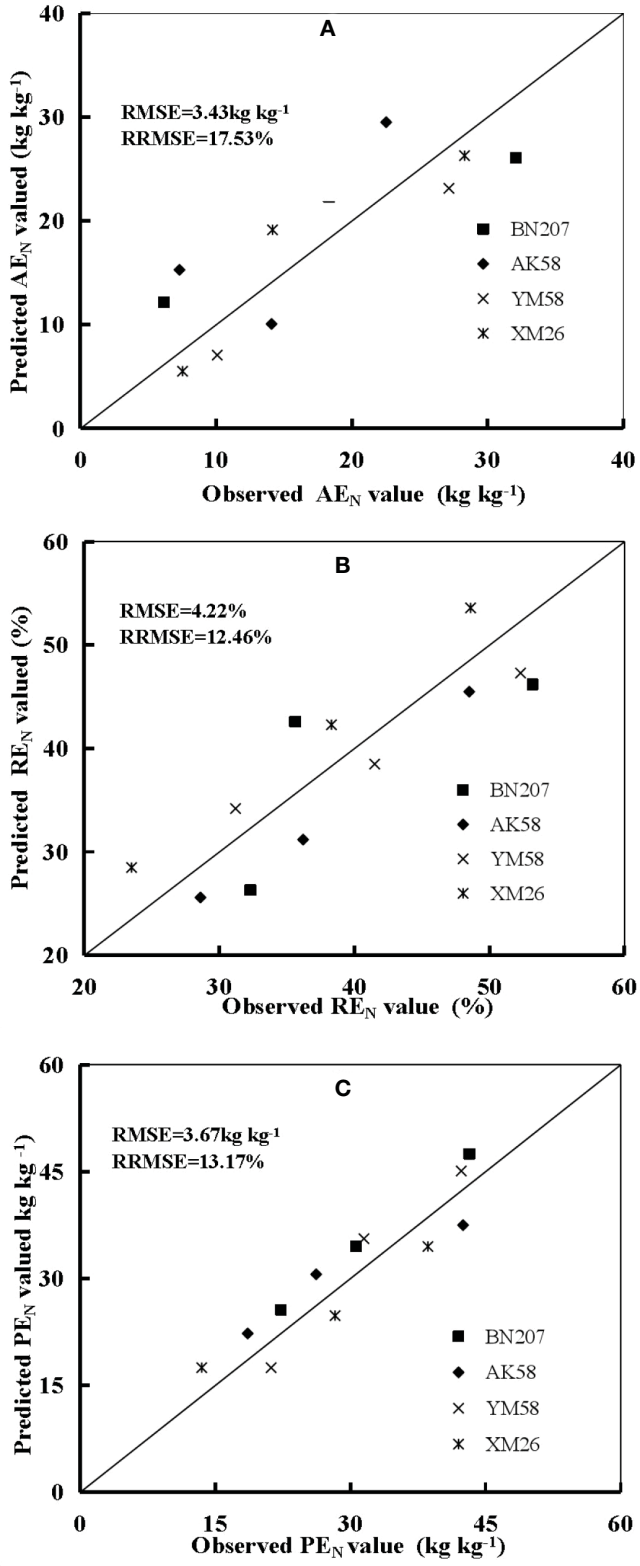
Comparison of the observed and predicted difference of AE_N_ **(A)**, RE_N_ **(B)**, and PE_N_ **(C)** values from 2016 to 2017 at Xinxiang and Jiaozuo.

Accumulated nitrogen deficit was positively correlated with AE_N_, RE_N,_ and PE_N_ for both cultivars during the 2016 to 2017 seasons at Xinxiang and Jiaozuo (**Figures 4**–**6**). The robustness of these relationships was validated using data acquired from an independent experiment from 2017 to 2018. The RMSE of 3.43 kg kg^−1^, 4.22%, and 3.67 kg kg^−1^ and RRMSE of 17.53%, 12.46%, and 13.17% were observed for AE_N_, RE_N,_ and PE_N_, respectively, while the values of R^2^ were 0.67, 0.73, and 0.85 for AE_N_, RE_N_, and PE_N_, respectively, indicating a good relationship between the observed and predicted AE_N_, RE_N_, and PE_N_ values (**Figure 7**).

## 4 Discussions

### 4.1 Effect of nitrogen rate on the growth of winter wheat

The nitrogen fertilizer application rate affects PNC during the growth period of winter wheat. PNC generally increases with increasing N application rates. Plants can accumulate excessive N nutrition in their organs (leaf and stem). A significant increase in plant dry matter accumulation and leaf area expansion with an increase in PNC under N-limiting conditions has been previously reported; however, excessive N application beyond the threshold level (non-limiting N conditions) poses negative impacts on plant growth (Yao et al., 2014; Zhao et al., 2014). Additionally, when soil N level is high, the result shows little increase in PNC or yield as N fertilizer increases in some studies (Feng et al., 2008). This might be because plants tend to absorb soil N, not fertilizer N (Grant et al., 1999; Lafond et al., 2008).

The increase in grain yield from N0 to N225 treatment in this study indicated that the winter wheat plant has very high N requirements (**Table 2**). However, the effect of N fertilizer on yield changes with site and year. An obvious change in yield under different soil types, climates, and cultivars in south Asia has been previously reported (Ladha et al., 2003). The change in winter wheat yield was mainly attributed to the change in yield components (the number of spikes per plant and grain weight per plant), and there was a significant effect of N application on the formation of yield components in winter wheat (Zhao et al., 2020). Excessive N application does not always warrant higher grain yield but decreases N use efficiency and poses negative impacts on the environment due to environmental loss of applied N up to ∼180 kg ha^−1^ yr^−1^ (Vitousek et al., 2009). The recommended N application rates of 120–170 kg N ha^−1^ to balance grain yield, NUE, and N loss in the North China Plain have been previously reported (Cui et al., 2009; Ma et al., 2019).

The effect of N application rates was more obvious on grain protein yield than grain protein content, which was attributed to the impacts of N fertilizer on grain yield and protein content. The grain protein content increased irrespective of N supply conditions; however, the increase in grain protein content was minor under N non-limiting treatments (Zhao et al., 2020). The non-significant effect of N fertilizer on TGW agreed with previous reports (Yan et al., 2019).

### 4.2 Effect of N application on accumulated nitrogen deficit, agronomic nitrogen use efficiency and its components, and their relationships

The variability in N_and_ with N rates and crop growth stages in this study was in consensus with a study on rice (Ata-Ul-Karim et al., 2013). N_and_ is a suitable index for in-season crop N deficit monitoring and can potentially be used to quantify the amount of supplemental N fertilization scheduled during crop growth. N_and_ has advantages over other indices such as PNC (Guillard et al., 2021), DM (Zhu et al., 2003), and chlorophyll concentration (Li et al., 2019) as it contains more information on crop growth conditions by integrating PNC with plant dry matter while diagnosing plant N status (Lemaire et al., 2008; Ziadi et al., 2008; Ata-Ul-Karim et al., 2017a).

Accumulated N deficit can be exploited for in-season estimation of crop N status and to fine-tune the supplemental N scheduling during the growth period as it is crop-specific, precise, and theoretically sound in relation to actual crop growth (Ata-Ul-Karim et al., 2017b). Being based on the N dilution theory, N_and_ is not only theoretically sound but also relevant agronomically as an N diagnostic tool to quantify the in-season crop N status. Results indicated that the winter wheat plant invested a higher proportion of dry matter in the stem (low organ N concentration) after Feekes stage 6 as compared to the leaf (high organ N concentration) to ensure optimal plant height and capture more light. Additionally, the decline in the N concentration of shaded leaves per unit leaf area contributes to optimizing plant N allocation in relation to light distribution for improving canopy photosynthesis (Lemaire et al., 2008).

The lower variability of AE_N_ and its components across cultivars and sites might be due to similar climatic conditions and management practices at both sites (**Figure 1**). The decrease in AE_N_ under N non-limiting treatments indicated that higher N fertilizer losses occurred under higher N application rate treatments. The variability of AE_N_ and PE_N_ between cultivars in this study was in consensus with inter- and intra-specific genetic variability for N fertilizer use among crop cultivars and species (Wu et al., 2006; Salim and Raza, 2020). The higher values of AE_N_ and PE_N_ of ZM27 indicated that a higher amount of grain yield was produced per unit of N fertilizer applied as compared to ZM22. Both cultivars showed non-significant differences in RE_N_, which indicates that AE_N_ and PE_N_ were mainly affected by the characteristics of the cultivars rather than RE_N_, which might be affected by external environments. Plant N uptake under field conditions is also affected by the high spatial and temporal heterogeneity in soil N availability (Finger et al., 2019). The lower RE_N_ at a high level of soil N was due to the lower absorption of fertilizer N by winter wheat plants. The accumulated NO^−3^-N in the soil poses an obvious effect on the N fertilizer use efficiency as a supplement of soil N has a very high replacement value for N fertilizer (Cassman et al., 2003). The proportion of N applied that is not absorbed by the plant under non-limiting N conditions increases the risk of N leaching due to the movement of applied N to deeper soil layers during the crop growth period (Villar-Mir et al., 2002).

The significantly positive relationships of N_and_ with AE_N_, RE_N,_ and PE_N_ indicated that N_and_ can be potentially applied to predict AE_N,_ RE_N,_ and PE_N_ during the crop growth period. N_and_ can also be used for the estimation of crop N requirements, which can be calculated as the difference between critical N accumulation and actual N accumulation (Eq. (2)). N_and_ has successfully differentiated the N sub-optimal optimal and supra-optimal N growth conditions (**Figure 3**), and N_and_ = 0 indicates that N nutrition is optimum, while N_and_ >0 indicates N deficiency and<0 points out luxury consumption (Ata-Ul-Karim et al., 2013). The relationships between these characteristics can also contribute to a better understanding of N fertilizer management for increasing agronomic N use efficiency. The high yield was the result of better exploitation of N, which is accompanied by lower AE_N_ with the increase in N application. RE_N_ describes the recovery efficiency of fertilizer N by the crop from the soil. RE_N_ can serve as an important parameter for strategizing precise and quantitative N application technologies. However, it is affected by various factors (soil type, N application, agro-climatic conditions, yield level, and cultivars; Conant et al., 2013). RE_N_ was approximately 40% in this study when N_and_ was equal to 0. A previous study reported that 40% of the RE_N_ of winter wheat could be considered a critical value in a high-yield field (Xu, 2010). RE_N_ will decrease with the decline of N_and_ when N_and_ is lower than 0 due to lower absorption of fertilizer N from the soil by the plant because of excessive N accumulation by the plant. PE_N_ is considered the most important component of AE_N_, as PE_N_ accounts for more of the variation in AE_N_ than RE_N_ (Dordas, 2008). PE_N_ generally remains relatively stable during the crop growth period and is little affected by grain yield. A cultivar with a higher RE_N_ should be selected for increasing AE_N_ (Novoa and Loomis, 1981; Raun and Johnson, 1999). Many trade-offs exist between the different components of AE_N_ as they are influenced by several factors.

## 5 Conclusions

Accumulated N deficits under different N rates demonstrated significant variation across sites, growth stages, and cultivars. N_and_ well distinguished the plant’s internal N status, which indicated that N_and_ has the potential to quantify the in-season crop N status of winter wheat. N_and_ was positively correlated with AE_N_ and its components. The validation results demonstrated that the newly developed models can accurately predict in-season AE_N_ and its components. The findings would assist in improving in-season N use efficiency by fine-tuning N scheduling decisions in winter wheat cultivation in intensive wheat cropping systems in China. However, more independent experiments under different agro-climatic conditions, crop management practices, and crop species are required to test the wide-scale applicability of N_and_ for in-season assessment of crop N status and N use efficiencies.

## Data availability statement

The original contributions presented in the study are included in the article/**Supplementary Material**. Further inquiries can be directed to the corresponding authors.

## Author contributions

BZ, LW, and SA conceived the idea and led the study design. BL and XM carried out the experiments, performed the analysis, and wrote the manuscript. BZ, LW, and SA reviewed and edited the manuscript. All authors listed have made a substantial, direct, and intellectual contribution to the work and approved it for publication.
