# Supplementary material for: In-season assessment of agronomic nitrogen use efficiency and its components in winter wheat using critical nitrogen dilution curve
Source: Front Plant Sci. 2023 Mar 15;14:1128799. doi: 10.3389/fpls.2023.1128799 (PMC10050426; doi:10.3389/fpls.2023.1128799)
Supplement: Supplementary file 1 [file Table_1.docx]

**Table S1 Description and units of the variables and parameters used in this paper**

| Name | Description | Unit |
| --- | --- | --- |
| N | Nitrogen | - |
| Nand | Accumulated N deficit | kg ha-1 |
| Nc | critical N | % |
| Nna | actual N accumulation | kg ha-1 |
| DM | dry matter | kg ha-1 |
| AEN | N fertilizer agronomy efficiency | kg kg-1 |
| PNC | Plant nitrogen concentration | % |
| TGW | Thousand grains weight | g |
| Gw | grain yield | kg |
| Nf | N fertilizer | kg |
| REN | N fertilizer recovery efficiency | % |
| PEN | N fertilizer physiological efficiency | kg kg-1 |

**Table S2 The ANOVA result of PNC from 2016 to 2017 season in ZM22 at Xinxiang**

| Growth stage | PNC of 2016-2017 ZM22 Xinxiang | | | | |
| --- | --- | --- | --- | --- | --- |
|  | N0 | N75 | N150 | N225 | N300 |
| Feekes 6 | 3.25 | 3.4 | 3.38 | 3.76 | 4.11 |
| Feekes11.5 | 1.1 | 1.2 | 1.35 | 1.61 | 1.87 |
| Significance | ** | ** | ** | ** | ** |

** indicated that *p* value <0.01.

**Table S3 The ANOVA result of PNC from 2016 to 2017 season in ZM27 at Xinxiang**

| Growth stage | PNC of 2016-2017 ZM27 Xinxiang | | | | |
| --- | --- | --- | --- | --- | --- |
|  | N0 | N75 | N150 | N225 | N300 |
| Feekes 6 | 2.48 | 3.27 | 3.19 | 3.35 | 3.46 |
| Feekes11.5 | 1.1 | 1.29 | 1.25 | 1.77 | 2.09 |
| Significance | ** | ** | ** | ** | ** |
